# Clinical outcomes of the PAUL glaucoma implant in primary open‐angle and pseudoexfoliative glaucoma eyes after failed glaucoma surgeries

**DOI:** 10.1111/aos.17581

**Published:** 2025-08-19

**Authors:** Constance Liegl, Leonie Bourauel, Benjamin Aretz, Diana Samarghitan, Wolfgang Walz, Michael Petrak, Frank G. Holz, Raffael Liegl, Karl Mercieca

**Affiliations:** ^1^ Department of Ophthalmology University of Bonn Bonn Germany; ^2^ Institute for Medical Biometry, Informatics and Epidemiology University of Bonn Bonn Germany; ^3^ Department of Ophthalmology Klinikum Dortmund, University Witten/Herdecke Dortmund Germany

**Keywords:** glaucoma surgery, PAUL glaucoma implant outcomes, PEX, POAG

## Abstract

**Purpose:**

This study assesses the PAUL® glaucoma implant (PGI) in primary open angle glaucoma (POAG) and pseudoexfoliative glaucoma (PEXG) after failed glaucoma surgery. Given PEXG's more aggressive nature, limited research exists on PGI outcomes in this subgroup. This study aims to compare PGI's efficacy and safety in both conditions.

**Methods:**

A retrospective cohort study including patients undergoing PGI surgery at the University Eye Hospital Bonn, Germany, from April 2021 to January 2024. Patients were enrolled in a database at the time of surgery, with follow‐up data collected at each visit. Success was defined according to four criteria, using intra‐ocular pressure (IOP) thresholds of ≤21 mmHg (Criterion A), ≤18 mmHg (Criterion B), ≤15 mmHg (Criterion C) and ≤12 mmHg (Criterion D).

**Results:**

Forty‐eight eyes of 48 patients were included. Qualified and complete success rates (95% CI) were 60.4% (45.8–72.9) and 93.8% (87.5–100) for Criterion A, 60.4% (45.8–72.9) and 85.4% (75–95.8) for Criterion B, 43.8% (29.2–56.3) and 58.3% (43.8–72.9) for Criterion C and 33.3% (20.8–47.9) and 35.4% (22.9–50) for Criterion D. Mean IOP decreased from 23.52 mmHg (7–45) to 11.65 mmHg (3–20 mmHg) with a mean reduction of 43.55% (0–82%) at 12 months, with a reduction in IOP‐lowering agents from 3.13 (1–5) to 0.44 (0–3). Postoperative complications occurred in 33.3% of cases. Approximately 6.3% required additional glaucoma procedures, leading to surgical failure. Regarding primary outcomes, no significant differences were observed for qualified or complete success rates between POAG and PEXG. Both groups had similar preoperative IOP and experienced significant postoperative reduction, with no statistically significant differences in IOP, medication use, visual outcomes, complications, revision rates or surgical failure at 12 months.

**Conclusions:**

In conclusion, the PGI demonstrated significant IOP reduction and decreased need for medication in both POAG and PEXG patients, with no major differences between the two groups at 12 months. The implant's overall safety and efficacy make it a viable surgical option for both conditions. Further studies are needed to assess long‐term outcomes and refine patient selection criteria.

## 1 INTRODUCTION

Glaucoma is a leading cause of irreversible vision loss, significantly impacting patients' quality of life. Treatment approaches vary based on disease severity, with surgical intervention recommended when medical therapy and laser treatments prove insufficient. Glaucoma drainage devices (GDDs) are commonly employed to manage chronic primary open‐angle glaucoma (POAG) and pseudoexfoliative glaucoma (PEXG), particularly in cases where initial filtering surgery has failed (Aref et al., 2017; Lee et al., 2020; Pereira et al., 2021). The PAUL® glaucoma implant (PGI) (Advanced Ophthalmic Innovations, Singapore) shares similarities with the Baerveldt glaucoma implant (BGI) in plate size and valveless design but features a narrower tube diameter. Early findings suggest that the PGI effectively lowers intra‐ocular pressure (IOP), reduces dependence on medication and has a favourable safety profile, with a notably low incidence of postoperative hypotony (José et al., 2021; Koh et al., 2020; Vallabh et al., 2021; Weber et al., 2023).

PEXG is characterized by fibrillary deposits in the anterior segment, particularly on the lens capsule and pupillary margin. Compared to POAG, it is associated with higher initial IOP, more severe visual field loss at diagnosis and a more rapid disease progression. Despite its clinical significance, research on PGI outcomes in PEXG remains limited. To date, only one study has specifically examined PGI results in PEXG or directly compared them to POAG. This study reported high success rates and minimal complications, with PEXG patients who had previously failed filtration surgery demonstrating promising outcomes (Olgun & Karapapak, 2024). However, no research has exclusively analysed PGI performance following failed glaucoma surgery, as most existing studies include a heterogeneous mix of glaucoma subtypes. This study aims to fill that gap by evaluating and comparing PGI outcomes in POAG and PEXG eyes after unsuccessful prior surgical intervention.

## 2 MATERIALS AND METHODS

### 2.1 Patients

The medical records of all patients who underwent PGI surgery at the Department of Ophthalmology at the University Hospital of Bonn, Germany, from April 2021 to March 2024 were reviewed. Patients were enrolled in a database at the time of surgery, and follow‐up data were prospectively collected during each postoperative visit. All patients underwent a comprehensive ophthalmic examination upon presentation, which included the assessment of best‐corrected visual acuity (BCVA) using the Snellen chart (converted to logMAR for statistical analysis), IOP measurement via Goldmann applanation tonometry, slit lamp biomicroscopy, fundus biomicroscopy and visual field testing using the Humphrey 24‐2 (Carl Zeiss Meditec, Inc., Dublin, CA) visual field test strategy. For each consecutive patient who received a PGI implant, the preoperative data collected included gender, age, type of glaucoma, BCVA, clinical features (e.g., IOP and anterior segment signs) and detailed follow‐up data on BCVA, IOP, visual fields, complications and postoperative glaucoma medications. Four success criteria (based on WGA guidelines) were applied, with IOP thresholds as follows:

- Criterion A: IOP ≤21 mmHg,
- Criterion B: IOP ≤18 mmHg,
- Criterion C: IOP ≤15 mmHg, and
- Criterion D: IOP ≤12 mmHg.

Success was classified as complete if achieved without glaucoma medications and as qualified if achieved with medication. PGI surgery was considered a failure in cases of persistent IOP below 6 mmHg and/or clinically significant hypotony at two consecutive visits after 3 months post‐surgery; the need for additional glaucoma surgery for uncontrolled IOP, PGI explantation or loss of light perception.

The primary outcome was the success rate based on the defined criteria. Secondary outcomes included IOP, BCVA, the number of IOP‐lowering medications, complications and details regarding polypropylene intraluminal stent removal. All analyses were performed on a de‐identified data set, and the study was approved by the Ethics Committee at the University Hospital Bonn (number: 458/21). The protocol adhered to the ethical standards of the 2000 Declaration of Helsinki, as confirmed by the institution's Human Research Committee.

### 2.2 Surgical technique

All surgeries were performed by experienced, fellowship‐trained glaucoma surgeons (KM + MP). The PGI is usually placed in the superotemporal quadrant but may be alternatively positioned superonasally or inferonasally when necessary. The superotemporal quadrant is regularly chosen since this location allows plate and bleb cover by the upper lid and also offers good surgical access. Conjunctival and Tenon's peritomy result in exposure of the superotemporal quadrant with a fornix‐based flap. Mitomycin‐C (MMC) at a concentration of 0.5 mg/mL is applied to the location of the PGI plate for 2 min. The plate is then placed under the recti muscles and sutured to the sclera with 9.0 nylon sutures, 10 mm away from the corneal limbus. A 6.0 polypropylene thread is inserted into the PGI tube. A 26G cannula is used to create a tunnel into the anterior chamber. The tube is shortened to the required length, placed through the tunnel and secured with a nylon 9/0 ‘box’ suture. A double‐layered patch graft (pericardium or fascia lata) is used to cover the tube (fibrin glue used for adhesion) and the prolene thread is usually placed in an inferior sub‐conjunctival pocket. The conjunctiva is closed with two 10/0 nylon corner slip knots and two 10/0 nylon mattress sutures.

The polypropylene stent is removed during the postoperative period when the IOP rises above the target level. While there was no fixed timeframe for removal, it is performed as needed; but usually never within the first 8 weeks after surgery.

### 2.3 Statistical analysis

Statistical analysis was performed using SPSS Statistics version 27.0.0 (IBM Corporation, New York). The paired sample *t*‐test was used to test differences between repeated measurements. Time‐to‐event data were analysed using Kaplan–Meier estimators, and subgroup comparisons were performed using the log‐rank test. Survival times are reported with 95% confidence intervals (CIs). For categorical variables, Fisher's exact test was used to compare distributions. For comparison between independent groups, *t*‐tests were used when data were normally distributed; otherwise, the non‐parametric Mann–Whitney *U* test was applied. All statistical tests were two‐sided; we considered *p* values <0.05 to be statistically significant.

## 3 RESULTS

Forty‐eight eyes of 48 patients who underwent PGI surgery after failed previous filtering glaucoma surgery with POAG or PEXG were included in this study. All patients were of Caucasian ethnicity. The mean age was 73.2 (34–89) years. Twenty‐nine eyes (60.4%) had POAG, and 19 eyes (39.6%) PEXG. All eyes (100%) had undergone previous glaucoma surgery, most commonly trabeculectomy (trab) (17 eyes, 35.4%) or cyclophotocoagulation (CPC) (17 eyes, 35.4%). Other patient characteristics are shown in Table 1. The mean preoperative IOP was 23.52 mmHg (7–45). All patients were using topical IOP‐lowering therapy with a mean of 3.13 agents (1–5 agents).

**TABLE 1:** Demographics and clinical characteristics of patients undergoing PGI surgery.

	*n* = 48 eyes
Gender	
Male/female	28 (58.2)/20 (41.7)
Ethnicity	
Caucasian	48 (100)
Age	
Mean (range)	73.2 (34–89)
Glaucoma type	
POAG	29 (60.4)
PEX glaucoma	19 (39.6)
Previous glaucoma surgery	
Yes	48 (100)
Which surgery	
Trabeculectomy	17 (35.4)
XEN stent implantation	6 (12.5)
Canaloplasty	6 (12.5)
Trabectome	5 (10.4)
CPC	17 (35.4)
Preserflo Microshunt	3 (6.3)
Deep sclerectomy	2 (4.2)
iStent	1 (2.1)
Needling after trab	5 (10.4)
Anaesthesia	
Local/general	15 (31.3)/33 (68.8)
Number of glaucoma drops	
Mean (range)	3.13 (1–5)
Acetazolamide	
Yes	11 (22.9)
Lens status	
Phakic. Pseudophakic	9 (18.7)/39 (81.3)
BCVA preoperatively (logMAR)	
Mean (range)	0.52 (0–2.3)
IOP preoperatively	
Mean (range)	23.52 (7–45)

Abbreviations: BCVA, best‐corrected visual acuity; IOP, intra‐ocular pressure; PEXG, pseudoexfoliative glaucoma; PGI, PAUL® glaucoma implant; POAG, primary open angle glaucoma.

**FIGURE 1:**
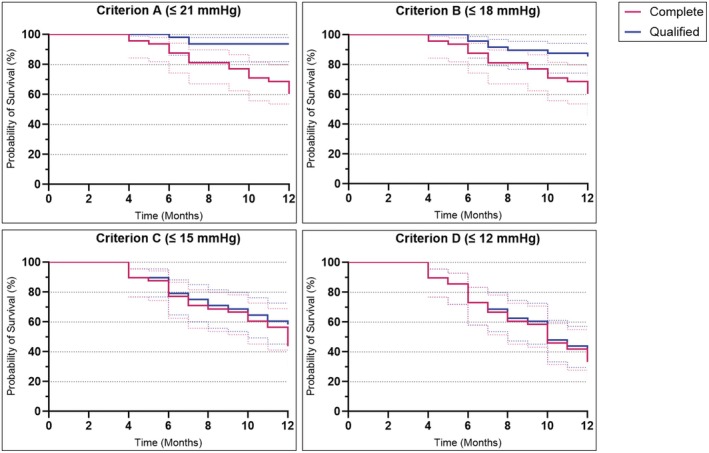
Kaplan–Meier curves with success rates (95% CI) 1 year after surgery.

Primary outcomes were related to the above‐mentioned success criteria. Complete and qualified success rates were 60.4% (95% CI 45.8–72.9) and 93.8% (95% CI 87.5–100) for Criterion A (IOP ≤21 mmHg), 60.4% (95% CI 45.8–72.9) and 85.4% (95% CI 75–95.8) for Criterion B (IOP ≤18 mmHg), 43.8% (95% CI 29.2–56.3) and 58.3% (95% CI 43.8–72.9) for Criterion C (IOP ≤15 mmHg) and 33.3% (95% CI: 20.8–47.9) and 35.4% (95% CI: 22.9–50) for Criterion D (IOP ≤12 mmHg) (Figure 1). Three eyes (6.3%) developed a failure event due to explanation of the PGI following persistent tube exposure despite surgical revision with a second patch graft (Table 5).

**FIGURE 2:**
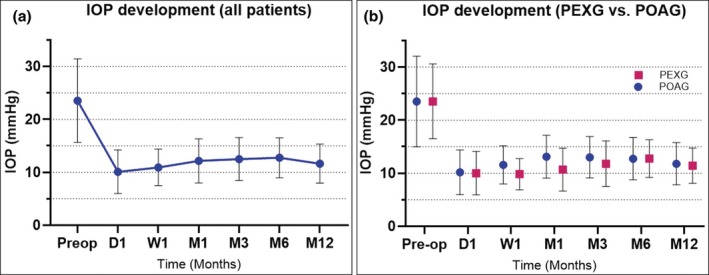
Development of intra‐ocular pressure (IOP) over 12 months of all patients (a) and comparison of patients with primary open angle glaucoma (POAG) and pseudoexfoliative glaucoma (PEXG) (b).

Following PGI implantation in our cohort, the mean IOP decreased from 23.52 mmHg (range 7–45) to 10.10 mmHg (range 4–25 mmHg) at day 1 postoperatively, 13.75 mmHg (range 3–23) at 6 months postsurgery and 11.65 mmHg (range 3–20 mmHg) at 12 months, the latter representing a mean reduction of 43.55% (range: 0%–82%) (Figure 2A). IOP values for other time points can be found in Table 2.

**TABLE 2:** Intra‐ocular pressure (IOP) and percentage reduction during the postoperative course.

Time point	IOP (range)	Percentage reduction
Maximum IOP	32.61 (17–59)	n/a
Preoperative	23.52 (7–45)	n/a
Day 1	10.10 (4–25)	52.24 (0–88)
Week 1	10.93 (5–18)	48.45 (0–79)
Month 1	12.15 (4–21)	43.51 (0–85)
Month 3	12.50 (4–21)	41.78 (0–76)
Month 6	12.75 (3–23)	39.29 (0–76)
Month 12	11.65 (3–20)	43.55 (0–82)

The mean number of IOP‐lowering agents was reduced from 3.13 (range 1–5) to 0.33 (range 0–3) and 0.44 (range 0–3) at 6 and 12 months, respectively. While 11 eyes (22.9%) needed systemic acetazolamide therapy preoperatively, no patient required it at any time point after surgery.

Mean BCVA was 0.52 logMAR (range 0–2.3) preoperatively and decreased after surgery to 0.96 logMAR (range 0.1–2.7) and 0.86 logMAR (range 0–2.3) on day 1 and week 1, respectively. However, this had deteriorated slightly in comparison to preoperative levels by 12 months after surgery, with a mean BCVA of 0.61 logMAR (range 0–2.3) at the 1‐year mark. This overall visual deterioration resulted from ocular comorbidities, especially due to one eye developing a central retinal vein occlusion with visual deterioration from 0.1 logMAR to light perception (Table 3).

**TABLE 3:** Best‐corrected visual acuity (BCVA) and pressure‐lowering eye drops.

Time point	BCVA	Number of agents
Preoperative	0.52 (0–2.3)	3.13 (1–5)
Day 1	0.96 (0.1–2.7)	0.10 (0–3)
Week 1	0.86 (0.1–2.3)	0.09 (0–2)
Month 1	0.72 (0–2.3)	0.13 (0–2)
Month 3	0.63 (0–2.3)	0.23 (0–3)
Month 6	0.67 (0–2.3)	0.33 (0–3)
Month 12	0.61 (0–2.3)	0.44 (0–3)

Postoperative complications were observed in 16 eyes (33.3%). Three eyes (6.3%) needed an intracameral injection of viscoelastic due to significant hypotony with AC shallowing. One eye (2.1%) developed aqueous misdirection, which resolved after a YAG‐laser zonuloiridohyaloidotomy. One eye (2.1%) had persisting corneal decompensation and underwent a Descemet membrane endothelial keratoplasty (DMEK). Six eyes (12.5%) developed tube exposure, which required conjunctival revision with an additional pericardial patch graft. Further complications are illustrated in Table 4. Three eyes (6.3%) needed additional glaucoma procedures that led to failure of the surgery (Table 5).

**TABLE 4:** Complications of PGI surgery.

Complications	Number of patients (%)
Complications overall	16 (33.3)
Corneascleral defect after previous trab and patchgraft	1 (2.1)
Hyphema	7 (14.6)
Hypotony with choroidal detachment	4 (8.3)
Injection of viscoelastic	3 (6.3)
Postoperative macular oedema and peripulbar corticosteroid injection	2 (4.2)
Aqueous misdirection resolved by YAG‐iridozonulohyaloidotomy	1 (2.1)
Fibrin response, resolved with topical therapy	1 (2.1)
Tube placed too anteriorly with endothelial cell loss and repositioning	1 (2.1)
Corneal decompensation and DMEK	1 (2.1)
Revision for tube erosion	6 (12.5)
One patient with tube erosion twice	1 (2.1)
Double vision (self‐compensating)	1 (2.1)
Retinal vein occlusion with visual deterioration from 0.1 logMAR to light perception	1 (2.1)

Abbreviations: DMEK, descemet membrane endothelial keratoplasty; PGI, PAUL® glaucoma implant.

**TABLE 5:** Additional glaucoma surgeries that lead to failure after PGI.

Procedures	Number of patients (%)
PGI‐explantation and re‐implantation in other quadrant	2 (4.2)
PGI explantation and Preserflo Microshunt	1 (2.1)

Abbreviation: PGI, PAUL® glaucoma implant.

An intraluminal stent in the form of a 6‐0 polypropylene thread was used in every case of this cohort. This was removed in 10 eyes (20.8%) after a mean time period of 6.40 months (range 1–26 months). Mean IOP before removal was 20.20 mmHg (range 14–25) and decreased to 10.0 mmHg (range 6–16). One eye needed an injection of viscoelastic due to low IOP after stent removal.

### 3.1 Comparison of eyes with POAG and PEXG

We conducted a comparison between eyes with PEXG and POAG, including 29 eyes with PEXG and 19 eyes with POAG. There were no significant differences in age (*p* = 0.051), gender (*p* = 0.084), ethnicity (*p* = 1.00), type of anaesthesia (*p* = 0.538) or lens status (*p* = 0.703), although the difference in age showed a trend towards significance. Further details and patient characteristics are shown in Table 6.

**TABLE 6:** Comparison of POAG and PEXG eyes.

	POAG	PEXG	*p*‐Value
Gender			0.084
Male/female	14 (48.3)/15 (51.7)	5 (26.3)/14 (73.7)	
Ethnicity			1.0
Caucasian	29 (100)	19 (100)	
Age			0.051
Mean (Jovine et al.)	70.58 (34–84)	77.21 (64–89)	
Anaesthesia			0.54
Local/general	8 (27.6)/21 (72.4)	7 (36.8)/12 (63.2)	
Acetazolamide			0.49
Yes	8 (27.6)	3 (16.7)	
Lens status			0.70
Phakic. Pseudophakic	6 (20.7)/23 (79.3)	3 (15.8)/16 (84.2)	
IOP development			
Preoperative	23.52 (7–45)	23.53 (12–38)	0.99
Day 1	10.17 (4–25)	10.0 (6–20)	0.88
Week 1	13.11 (5–26)	10.67 (5–14)	0.10
Month 1	13.00 (5–21)	11.78 (4–20)	0.06
Month 3	13.04 (4–20)	11.78 (6–21)	0.31
Month 6	12.74 (3–23)	12.76 (6–18)	0.98
Month 12	11.79 (3–20)	11.42 (5–20)	0.86
BCVA development			
Preoperative	0.49 (0–2.3)	0.56 (0–1.7)	0.64
Day 1	0.88 (0.1–2.3)	1.1 (0.1–2.7)	0.29
Week 1	0.74 (0.1–2.3)	1.03 (0.1–2.3)	0.21
Month 1	0.73 (0–2.3)	0.50 (0–2.3)	0.21
Month 3	0.71 (0–2.3)	0.50 (0–1.0)	0.22
Month 6	0.61 (0–2.3)	0.77 (0–2.3)	0.39
Month 12	0.62 (0–2.3)	0.78 (0–2.3)	0.78
Number of IOP lowering drops			
Preoperative	3.28 (1–5)	2.89 (1–4)	0.22
Day 1	0.17 (0–2)	0.0 (0–0)	0.24
Week 1	0.14 (0–2)	0 (0–0)	0.22
Month 1	0.17 (0–2)	0.06 (0–1)	0.39
Month 3	0.30 (0–3)	0.11 (0–2)	0.37
Month 6	0.35 (0–2)	0.29 (0–3)	0.79
Month 12	0.59 (0–3)	0.21 (0–2)	0.13
Complications/revisions			
Complications: yes	10 (34.5)	6 (33.3)	0.59
Revision: yes	3 (10.3)	3 (15.8)	0.57
Failure due to additional glaucoma surgery	2 (6.9)	1 (5.3)	0.59

Abbreviations: BCVA, best‐corrected visual acuity; IOP, intra‐ocular pressure; PEXG, pseudoexfoliative glaucoma; PGI, PAUL® glaucoma implant; POAG, primary open angle glaucoma.

Regarding primary outcomes, no significant differences were observed for qualified or complete success rates. At 1 year, complete and qualified success rates for Criterion A (IOP ≤21 mmHg) were 58.6% and 96.6% for POAG compared to 63.2% and 89.5% for PEXG (*p* = 0.761 and *p* = 0.554, respectively). For Criterion B (IOP ≤18 mmHg), success rates were 58.6% and 82.8% for POAG and 63.2% and 89.5% for PEXG (*p* = 0.753 and *p* = 0.687, respectively). For Criterion C (IOP ≤15 mmHg), they were 41.4% and 55.2% for POAG and 47.7% and 63.2% for PEXG (*p* = 0.770 and *p* = 0.766, respectively), while they were 34.5% and 34.5% for POAG and 31.6% and 36.8% for Criterion D (IOP ≤12 mmHg), respectively (*p* = 0.100 and *p* = 0.835).

Mean preoperative IOP was similar between both groups (PEXG with 23.52 mmHg vs. POAG with 23.53 mmHg, *p* = 0.99) (Figure 2B). Both groups showed substantial IOP reduction postoperatively. On day 1, mean IOP dropped to 10.17 mmHg for POAG and 10.0 mmHg for PEXG. Although IOP remained slightly higher in the POAG group at 3 and 6 months, this difference was not statistically significant. At 12 months post‐surgery, IOP was 11.79 mmHg for POAG and 11.42 mmHg for PEXG (*p* = 0.87).

There were no significant differences regarding need for IOP‐lowering drops or BCVA in both groups (Table 6). Furthermore, there were no differences in the number of complications (*p* = 0.596), revision rates (*p* = 0.569) and failure rates (*p* = 0.594).

## 4 DISCUSSION

The PGI is an innovative device that has demonstrated a promising ability to lower IOP (Tan et al., 2022). However, most studies have either included a heterogeneous mix of glaucoma subtypes or focused on specific conditions, such as uveitic glaucoma (Richardson et al., 2024) or following vitreoretinal (VR) surgery (Karapapak & Olgun, 2024; Weber et al., 2025). To date, no study has exclusively examined the outcomes of PGI in patients with POAG or PEXG following failed glaucoma surgery, and only one study has directly compared these two subtypes.

Our study demonstrated a significant reduction in IOP and the need for pressure‐lowering eye drops following PGI implantation in patients with a history of unsuccessful glaucoma surgery. IOP decreased from a preoperative mean of 23.52 mmHg (range 7–45 mmHg) to 10.10 mmHg (4–25 mmHg) on the first postoperative day, stabilising at 11.65 mmHg (3–20 mmHg) at 12 months, reflecting a mean reduction of 43.55% (0%–82%). To date, no prior study has exclusively investigated PGI outcomes in eyes following unsuccessful glaucoma surgery, making direct comparisons to similar cohorts unavailable. However, our findings align with broader PGI literature. Comparing IOP values and success rates across different glaucoma studies poses challenges, as success criteria vary; some rely solely on absolute IOP thresholds, while others incorporate percentage reductions. For this reason, direct comparisons to the PGI studies, as by José et al. (2021), must be interpreted with caution. In our study, success rates were notably high based on our predefined criteria. Using Criterion A (IOP ≤21 mmHg), the success rate was 94% for qualified success and 60% for complete success. Under Criterion B (IOP ≤18 mmHg), success rates were 85% and 60%, respectively. Similar to our approach, Vallabh et al. assessed success using IOP thresholds, reporting a 90.1% success rate for IOP levels between 5 and 21 mmHg—closely mirroring our findings of 94% success within the same range. The complete 1‐year success rate in their study was 38.4%, lower than our 60%, potentially due to the higher proportion (23%) of African American or sub‐Saharan African patients, who are known to have an increased risk of surgical failure due to subconjunctival scarring (Vallabh et al., 2021). Koh et al. (2020) reported a qualified success rate of 93.2% and a complete success rate of 68.9%, also aligning closely with our data. A prior study from our group, which included a broader range of glaucoma subtypes with a high proportion of secondary glaucoma cases, reported a comparable qualified success rate of 95.6%, though a higher complete success rate of 73.3% (Weber et al., 2023). Overall, our findings underscore that PGI achieves favourable success rates, effectively maintaining IOP below 21 and 18 mmHg, even in patients with a history of failed glaucoma surgery.

At 12 months, IOP in our cohort decreased from 23.52 mmHg (7–45 mmHg) to 11.65 mmHg (3–20 mmHg). These results are consistent with previous studies on PGI outcomes: Koh et al. (2020) reported an IOP reduction from 34.3 to 13.2 mmHg after 1 year, whereas José et al. (2021) observed a decrease from 31.4 to 12.5 mmHg over the same period. However, it is noteworthy that our study's baseline IOP was lower, leading to a comparatively smaller percentage reduction. This discrepancy likely arises from differences in patient populations, as secondary glaucoma subtypes—such as neovascular glaucoma or post‐vitrectomy glaucoma—often present with markedly elevated IOP levels. In contrast, our cohort focused solely on POAG and PEXG following failed surgical intervention, where disease progression is already significant at IOP levels in the mid‐20 mmHg range.

Our study demonstrated a comparable IOP‐lowering efficacy between POAG and PEXG, with no significant differences in complication rates, failure rates, or the need for IOP‐lowering medication. Olgun et al. similarly evaluated PGI outcomes in PEXG and compared them to POAG, analysing a cohort of 39 PEXG and 29 POAG eyes (Olgun & Karapapak, 2024). In their study, PEXG patients were significantly older than those with POAG, a finding not replicated in our cohort. Given the more aggressive nature of PEXG, it is often assumed that patients would require surgical intervention earlier than those with POAG, potentially resulting in lower preoperative IOP. However, this assumption was not reflected in the findings of Olgun et al. They also found no significant differences in success rates between the two subtypes; though at 12 months, a slightly higher proportion of PEXG patients achieved an IOP ≤21 mmHg compared to POAG (97.4% vs. 86.2%). At the 1‐year follow‐up, IOP remained comparable between PEXG and POAG (13.7 mmHg vs. 14.8 mmHg, respectively). However, their reported IOP values were slightly higher than those observed in our study.

To date, no other studies have directly compared PGI outcomes between PEXG and POAG. Moreover, literature on the efficacy of other GDDs in these subtypes remains scarce. Un et al. evaluated AGV implantation and found that, at the final postoperative visit, PEXG eyes achieved a lower IOP than POAG eyes (11.6 mmHg vs. 14.5 mmHg). Their study concluded that AGV implantation was highly successful in both subtypes, with PEXG patients reaching lower IOP levels at the last follow‐up (Un & Imamoglu, 2024). Similarly, Tojo et al. analysed BGI outcomes in PEXG and POAG, reporting a reduction in IOP from 30.0 to 10.8 mmHg at 1 year, with no significant differences between the two subtypes (Tojo & Hayashi, 2021).

Comparative studies on other surgical approaches for PEXG and POAG suggest that many alternatives are less effective for PEXG (Rabiolo et al., 2024; Wakuda et al., 2024; Weber et al., 2024). This is often attributed to the accumulation of PEX material on the anterior lens capsule, pupil margin, lens zonules and anterior chamber angle, raising the possibility that it may also deposit at the surgical site, potentially leading to less favourable outcomes. However, our data suggest that the PGI implantation as a GDD provides an effective IOP‐lowering solution for patients with refractory glaucoma after unsuccessful surgery. Unlike findings from other surgical modalities, we did not observe a higher risk of surgical failure in PEXG patients, indicating that PGI is an equally effective option for both PEXG and POAG.

This study has several limitations, primarily its retrospective design. Although data were prospectively collected at each consecutive patient visit, study‐specific appointments were not scheduled, leading to some loss to follow‐up. Despite proactive efforts, including telephonic outreach to patients and local ophthalmologists, we were unable to obtain complete 3‐year data for all patients who underwent PGI implantation in the initial months after its introduction. This inevitably introduces a selection bias.

A prospective, randomized clinical trial would be valuable in the future to compare different surgical techniques and provide more robust conclusions. Additionally, as this study was non‐comparative, it does not offer insights into how PGI performs relative to other established GDDs in eyes with POAG and PEXG.

## 5 CONCLUSION

This retrospective, comparative study suggests that PGI effectively lowers IOP and reduces the need for pressure‐lowering medications in patients after unsuccessful glaucoma surgery, in both POAG and PEXG patients. The procedure maintains a strong safety profile, with no vision‐threatening complications observed in either group.

## AUTHOR CONTRIBUTIONS

CL, LB, DS, WW and MP: collected data. CL, LB and RL: analysed data. CL, LB, FGH, RL and KM: wrote the manuscript. All authors approved the final version of the manuscript.

## FUNDING INFORMATION

No funding was received for this research. CL received funding from the Ernst‐und‐Berta‐Grimmke‐Stiftung.

## CONFLICT OF INTEREST STATEMENT

CL, LB, WW, DS and MP certify that they have no affiliations with or involvement in any organization or entity with any financial interest (such as honoraria; educational grants; participation in speakers' bureaus; membership, employment, consultancies, stock ownership or other equity interest and expert testimony or patent‐licensing arrangements) or non‐financial interest (such as personal or professional relationships, affiliations, knowledge or beliefs) in the subject matter or materials discussed in this manuscript. KM has received honoraria or research grants from the following: AOI (C, H, R) and FCI (H) but none of this is directly related to the current study.

## ETHICS STATEMENT

This study protocol was reviewed and approved by ethics review board at the University of Bonn, ‘Ethikkommission der Universität Bonn’, approval number 458/21.

## CONSENT STATEMENT

No written informed consent was obtained from participants to participate in the study. Due to the retrospective character of this study and since used data does not allow to identify patients, a waiver of informed consent was obtained by the local ethics committee. The ethics review board at the University of Bonn, ‘Ethikkommission der Universität Bonn’ gave a waiver under the reference number 258/21. The research was conducted ethically in accordance with the World Medical Association Declaration of Helsinki.

## Data Availability

All data sets generated during and/or analysed during the current study are available from the corresponding author upon reasonable request.
